# Differential diagnosis of thyroid nodules using heterogeneity quantification software on ultrasound images: correlation with the Bethesda system and surgical pathology

**DOI:** 10.1038/s41598-024-60881-2

**Published:** 2024-05-04

**Authors:** Young Jae Ryu, Jin Woong Kim, Sang Chun Park, Young Hoe Hur, Hyung Joong Kim, Tae-Hoon Kim

**Affiliations:** 1 Department of Surgery, Chonnam National University Medical School, Chonnam National University Hwasun Hospital, 322 Seoyang-ro, Hwasun-eup, Hwasun-gun, Jeonnam, 58128 Republic of Korea; 2 Department of Radiology, Chosun University College of Medicine, Chosun University Hospital, Gwangju, 61452 Republic of Korea; 3 Medical Science Research Institute, Kyung Hee University Hospital, 23 Kyungheedae-ro, Dongdaemun-gu, Seoul, 02447 Republic of Korea

**Keywords:** Ultrasonography, Translational research

## Abstract

Ultrasonography (US)-guided fine-needle aspiration cytology (FNAC) is the primary modality for evaluating thyroid nodules. However, in cases of atypia of undetermined significance (AUS) or follicular lesion of undetermined significance (FLUS), supplemental tests are necessary for a definitive diagnosis. Accordingly, we aimed to develop a non-invasive quantification software using the heterogeneity scores of thyroid nodules. This cross-sectional study retrospectively enrolled 188 patients who were categorized into four groups according to their diagnostic classification in the Bethesda system and surgical pathology [II-benign (B) (n = 24); III-B (n = 52); III-malignant (M) (n = 54); V/VI-M (n = 58)]. Heterogeneity scores were derived using an image pixel-based heterogeneity index, utilized as a coefficient of variation (CV) value, and analyzed across all US images. Differences in heterogeneity scores were compared using one-way analysis of variance with Tukey’s test. Diagnostic accuracy was determined by calculating the area under the receiver operating characteristic (AUROC) curve. The results of this study indicated significant differences in mean heterogeneity scores between benign and malignant thyroid nodules, except in the comparison between III-M and V/VI-M nodules. Among malignant nodules, the Bethesda classification was not observed to be associated with mean heterogeneity scores. Moreover, there was a positive correlation between heterogeneity scores and the combined diagnostic category, which was based on the Bethesda system and surgical cytology grades (R = 0.639, p < 0.001). AUROC for heterogeneity scores showed the highest diagnostic performance (0.818; cut-off: 30.22% CV value) for differentiating the benign group (normal/II-B/III-B) from the malignant group (III-M/V&VI-M), with a diagnostic accuracy of 72.5% (161/122). Quantitative heterogeneity measurement of US images is a valuable non-invasive diagnostic tool for predicting the likelihood of malignancy in thyroid nodules, including AUS or FLUS.

## Introduction

The prevalence of thyroid nodules in clinical practice has increased considerably owing to the widespread use of high-resolution imaging techniques and the generalization of health examinations^1^. Ultrasonography (US) is widely regarded as an effective and non-invasive modality for detecting and assessing thyroid nodules. The principal goal of US in evaluating thyroid nodules is to determine when cytology is necessary and whether these are benign or malignant. The Thyroid Imaging, Reporting and Data System (TI-RADS), which considers factors such as composition, echogenicity, shape, margin, echogenic foci, and size, has become widely used for further management^2^. US-guided fine-needle aspiration cytology (FNAC) is an essential procedure for the management of thyroid nodules, providing guidance on triaging patients who require further evaluation or surgery^3^. Approximately 60–70% of thyroid FNAC specimens are classified as benign, while approximately 20–30% are divided into three categories: suspicious for follicular neoplasm, suspicious for malignancy, and malignant. The remaining 5–10% of thyroid FNAC samples are classified as atypia of undetermined significance (AUS) or a follicular lesion of undetermined significance (FLUS)^4^.

The results of FNAC can be subject to variations across pathologists and institutions. Diagnostic uncertainty with AUS/FLUS represents a controversial issue and poses a challenge for management. Conventional recommendations for AUS/FLUS thyroid nodules are diagnostic lobectomy or repetitive FNAC. However, a majority of AUS/FLUS thyroid nodule specimens were revealed to be benign on surgical examination^5^. Repetitive FNAC does not necessarily guarantee a higher malignancy detection rate and may induce patient distress and complications^6^. Core needle biopsy (CNB) could be applied for AUS/FLUS thyroid nodules as an additional diagnostic approach; however, similar malignancy detection rates have been reported and the size of the thyroid nodule that qualifies for CNB might be restrictive compared with that for FNAC^7,8^. Molecular testing could be influenced by the size or sonographic features of the thyroid nodule, as well as regional economic status^9,10^. Therefore, it would be useful to resolve diagnostic uncertainty based on quantitative information that accurately reflects the intrinsic characteristics of thyroid nodules.

Recently, texture analysis has emerged as a viable diagnostic tool for thyroid nodules.^11^ The technique involves the use of computer-aided algorithms to extract texture features from US images^12^. Texture features that are commonly used in thyroid nodule analysis include entropy, homogeneity or heterogeneity, and contrast and can be used to differentiate between benign and malignant nodules based on factors such as size, shape, and internal composition. Since the introduction of a computer-aided diagnostic system for several solid malignancies, US image analysis of thyroid nodules using machine-learning algorithms has shown similar accuracy, sensitivity, and specificity to those of experienced radiologists for classifying benign and malignant cases^13,14^. Recently, Kim et al. proposed a novel scoring method for thyroid nodules using multiparametric photoacoustic analysis and the American Thyroid Association’s clinical practice guidelines^15^. However, for routine clinical use, it was necessary to test the reproducibility or the inter-physician variations of the method. The intrinsic features of thyroid nodules, such as cytopathologic atypia, architectural atypia, and necrosis, can affect the interpretation of FNAC. Several imaging studies have reported that the non-invasive assessment of heterogeneity within organs, such as using histograms, kurtosis, skewness, and coefficient of variation based on image signal intensities, differentiates the progression of liver diseases and brain lesions^16,17^. Thus, there is an unmet need for a new quantitative evaluation index to determine the malignancy of thyroid nodules. Hence, we developed a non-invasive quantification software that can analyze parenchymal heterogeneity scores in thyroid US images and evaluated its clinical applicability in distinguishing uncertain nodules such as AUS/FLUS.

## Results

### Patient characteristics

The FNAC was classified into six categories using the Bethesda scoring system as follows: Class I = nondiagnostic or unsatisfactory, Class II = benign (B), Class III = AUS or FLUS, Class IV = follicular neoplasm or suspicious for follicular neoplasm, Class V = suspicious for malignancy, and Class VI = malignant (M). Table 1 presents the patient demographics and characteristics of the thyroid nodules in this study. Among the 188 enrolled patients, 43 were males, and the mean age was 47.4 years. The final diagnoses included nodular hyperplasia (n = 33), follicular adenoma (n = 35), Hürthle cell adenoma (n = 1), non-invasive follicular neoplasm with papillary-like nuclear features (n = 7), papillary thyroid carcinoma (PTC) follicular variant (n = 12), and PTC (n = 100).

**Table 1:** Characteristics of the enrolled patients with thyroid nodules based on the Bethesda system and surgical pathology.

	Total (n = 188)	II-B (n = 24)	III-B (n = 52)	III-M (n = 54)	V/VI-M (n = 58)	*p*-value*^,†^	Patial eta squared (η^2^)
Age (year)	47.4 ± 12.7	44.8 ± 14.1	48.2 ± 14.7	47.0 ± 11.0	48.1 ± 11.7	*0.693	0.008
Male:female (n)	43:145	4:20	16:36	7:47	16:42	^†^0.107	0.032
Pathology* (n)							
Nodular hyperplasia	33	12	21	0	0		
Follicular adenoma	35	11	24	0	0		
Hürthle cell adenoma	1	0	1	0	0		
NIFTP	7	1	6	0	0		
PTCFV	12	0	0	12	0		
PTC	100	0	0	42	58		
TSH level (mIU/L)	2.07 ± 1.29	1.98 ± 1.55	1.91 ± 1.10	2.17 ± 1.31	2.15 ± 1.31	^†^0.997	< 0.001
Pre TSH ≤ 2	110	14	31	31	34		
Pre TSH > 2	78	10	21	23	24		
Nodule size at US (mm)	17.2 ± 12.6	38.3 ± 9.7	22.1 ± 11.9	9.9 ± 6.4	10.9 ± 4.0	* < 0.001^abcde^	0.578

B: benign; M: malignant, NIFTP: non-invasive follicular thyroid neoplasm with papillary like nuclear features, PTC: papillary thyroid carcinoma. PTCFV: papillary thyroid carcinoma follicular variant, TSH: thyroid-stimulating hormone.

Pathology data are presented as the number of patients.

Effect size was calculated as the partial eta squared value (η^2^) for measuring the difference between the means of four groups. Refer to effect sizes as small (η^2^ = 0.01), medium (η^2^ = 0.06), and large (η^2^ = 0.14).

*The differences among three fibrosis groups were analyzed using one-way ANOVA with Tukey’s post-hoc test as follows: ^a^II-B vs. III-B; ^b^II-B vs. III-M; ^c^II-B vs. V/VI-M; ^d^III-B vs. III-M; ^e^III-B vs. V/VI-M; ^f^III-M vs. V/VI-M.

† The differences among fibrosis groups in pathology data were analyzed using Pearson’s chi-square test.

**Figure 1:**
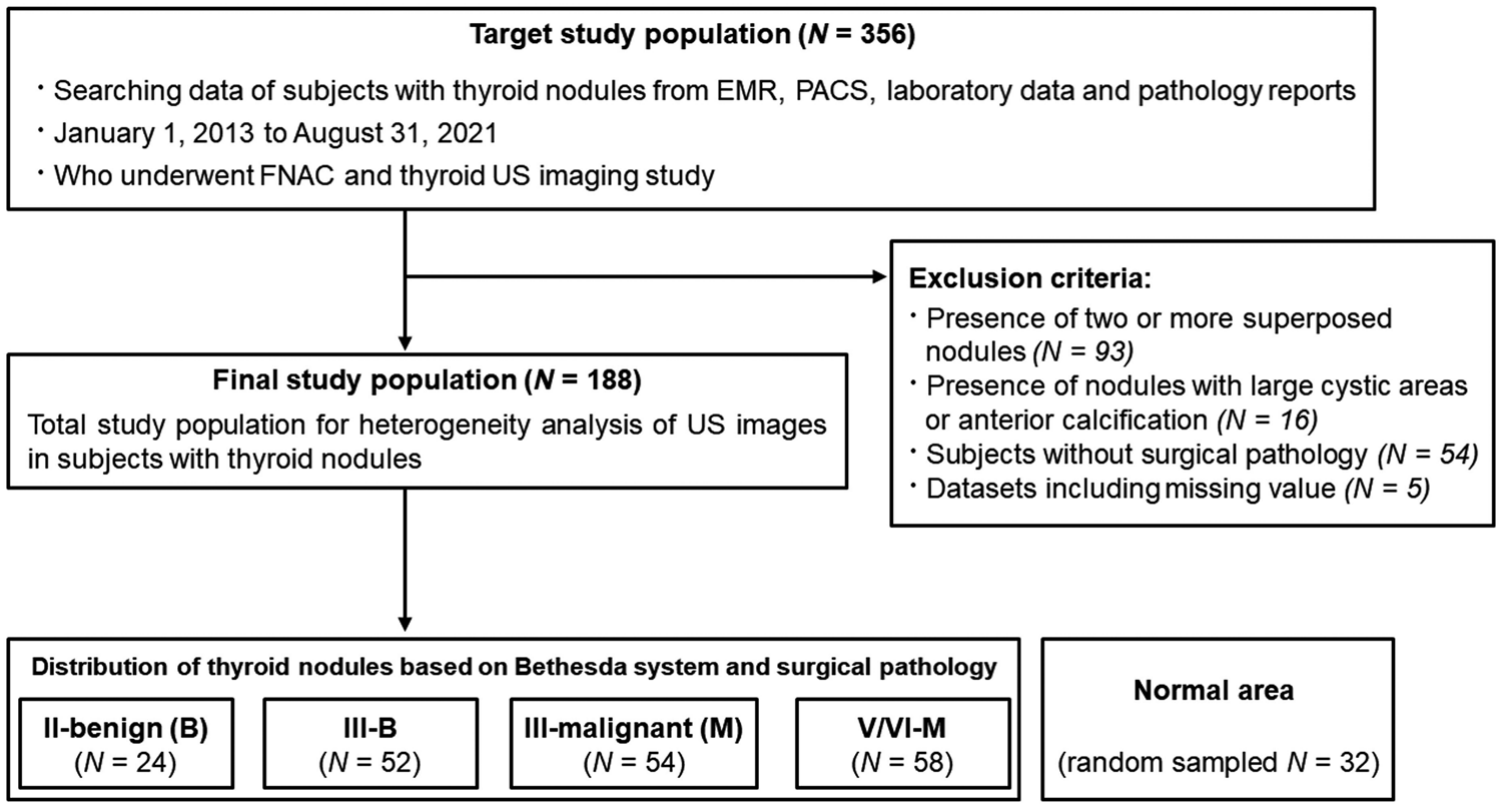
Flowchart of the inclusion of the study population. Compared to thyroid nodules, normal areas from 32 randomly sampled patients (17%) were measured using heterogeneity scores. B, benign; M, malignant; EMR, electronic medical records; PACS, picture archiving and communication system; US, ultrasonography; FNAC, fine needle aspiration cytology.

The combined diagnostic categories of the Bethesda system and surgical pathology were divided into four groups: II-B (n = 24), III-B (n = 52), III-M (n = 54), and V/VI-M (n = 58) (Fig. 1). No association was observed between thyroid-stimulating hormone (TSH) levels in any group. The maximum diameter of benign thyroid nodules tended to be larger as shown by US (Table 1).

### Heterogeneity measurements of thyroid nodules in US images according to the Bethesda system and surgical pathology

**Figure 2:**
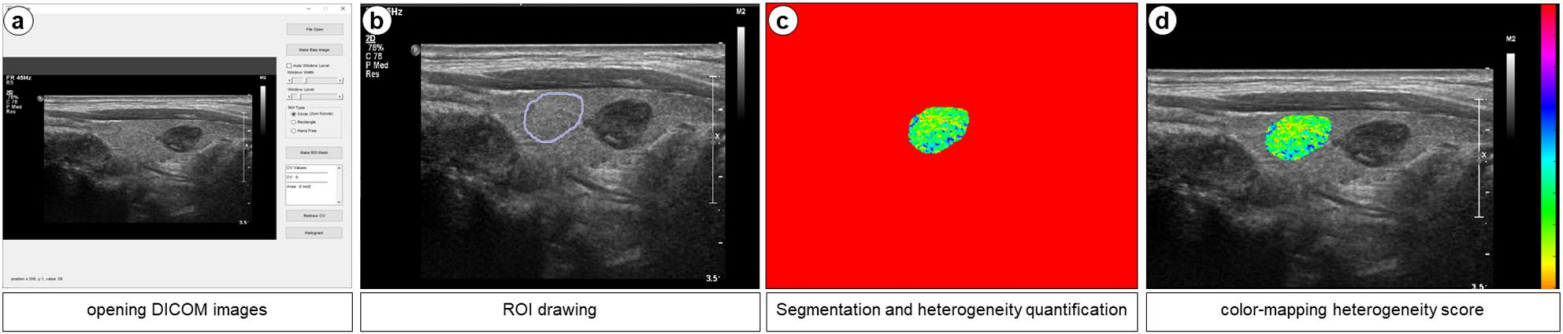
Diagram for assessing heterogeneity in the representative normal region using thyroid nodule ultrasonography images and the graphical user interface (GUI) with a sample US image on a quantification software. The processing procedures for assessing the heterogeneity were as follows: (**a**) opening US DICOM images, (**b**) manual drawing for region of interest (ROI) boundary detection, (**c**) region segmentation on US image and heterogeneity quantification of the segmented US image, and (**d**) color-mapping of heterogeneity scores. The manually drawn normal ROI is placed within the surrounding thyroidal parenchyma because the abnormal thyroid nodule is equal in size.

**Figure 3:**
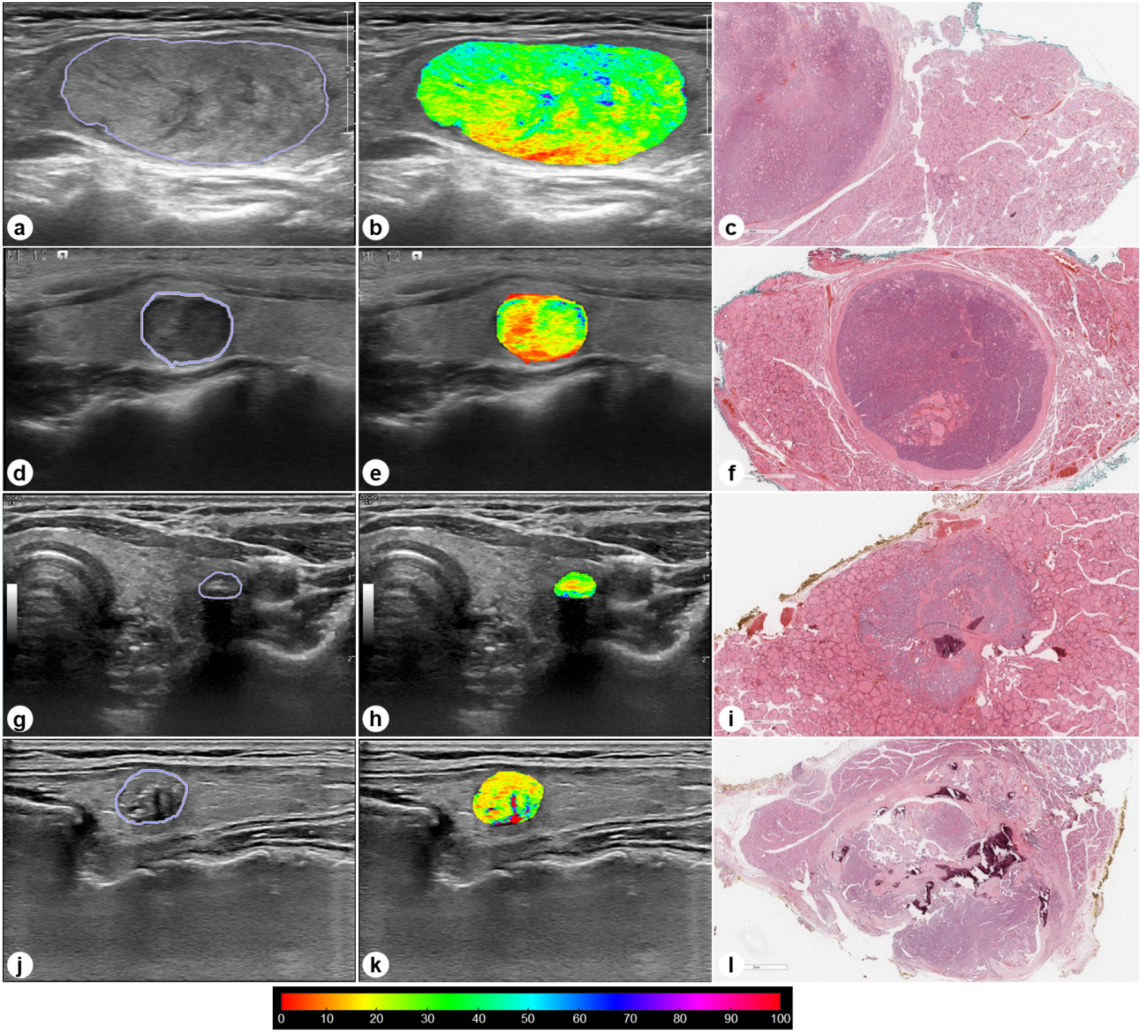
Representative thyroid US images, heterogeneity maps, and pathological specimens (hematoxylin and eosin staining) of II-benign (B) (**a–c**), III-B (**d–f**), III-malignant (M) (**g–i**), and V/VI-M (**j–l**). The heterogeneity scores for II-B (**b**), III-B (**e**), III-M (**h**), and V/VI-M (**k**) were 19.7%, 31.4%, 41.7%, and 45.1%, respectively.

**Figure 4:**
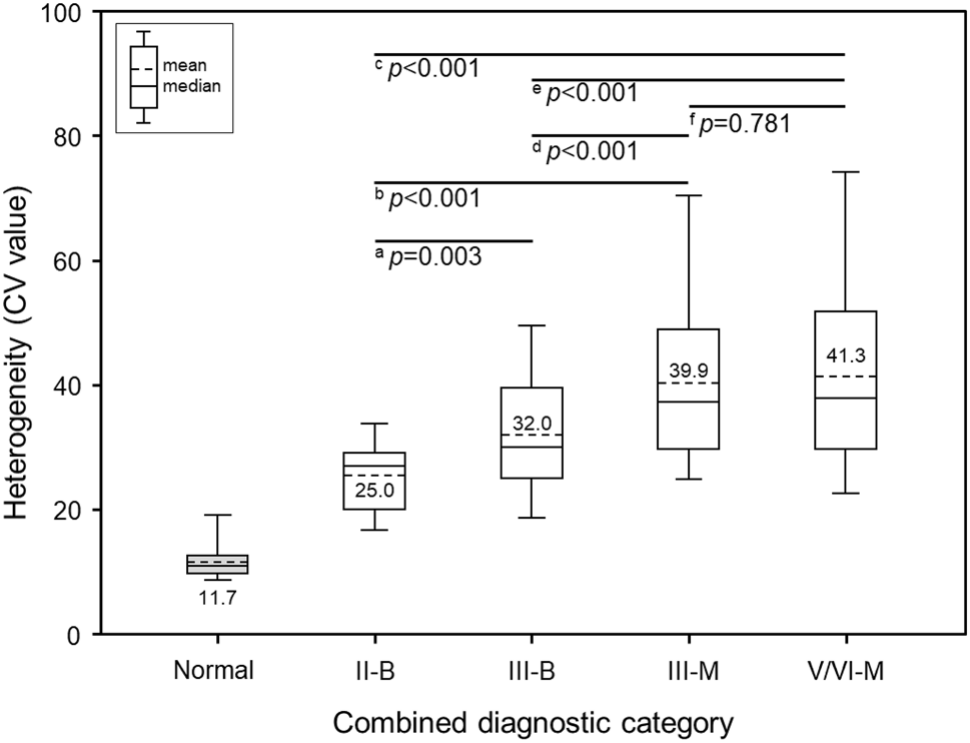
Boxplots including multiple comparisons show heterogeneity scores for the pathological grades. Differences between each group were analyzed using one-way ANOVA with Tukey’s post-hoc test: superscript a: II-B vs. III-B; superscript b: II-B vs. III-M; superscript c: II-B vs. V/VI-M; superscript d: III-B vs. III-M; superscript e: III-B vs. V/VI-M; superscript f: III-M vs. V/VI-M. The short dashed line indicates the mean heterogeneity score in each group, and the heterogeneity score in normal regions without nodules refers to the reference score for comparison.

Figure 2 shows a diagram for assessing heterogeneity in the representative normal region using thyroid nodule US images. Figure 3 shows representative thyroid US images and the selection of region-of-interests (ROIs) for heterogeneity quantification on heterogeneity maps for the four groups. The mean heterogeneity scores were significantly different among the four groups (analysis of variance (ANOVA); *p* < 0.001) (Table 2). In multiple comparisons, the mean heterogeneity scores in the four groups were significantly different, except for those in the comparison between III-M and V/VI-M, which were not statistically significant. Specifically, the results were as follows: II-B vs. III-B (*p* = 0.003), II-B vs. III-M (*p* < 0.001), II-B vs. V/VI-M (*p* < 0.001), III-B vs. III-M (*p* < 0.001), III-B vs. V/VI-M (*p* < 0.001), and III-M vs. V/VI-M (*p* = 0.781) (Fig. 4).

**Table 2:** Comparison of heterogeneity scores among the four groups of thyroid nodules based on the Bethesda system and surgical pathology.

	Normal area	II-B (n = 24)	III-B (n = 52)	III-M (n = 54)	V/VI-M (n = 58)	*p*-value*	Patial eta squared (η^2^)
Heterogeneity (%)	11.74 ± 2.84	25.02 ± 5.82	32.04 ± 10.34	39.93 ± 14.79	41.30 ± 16.12	< 0.001^abcde^	0.180

Data presented as mean ± SD.

The heterogeneity score in normal area was calculated as the reference level for comparison with the heterogeneity scores of thyroid nodules.

Effect size was calculated as the partial eta squared value (η^2^) for measuring the difference between the means of four groups. Refer to effect sizes as small (η^2^ = 0.01), medium (η^2^ = 0.06), and large (η^2^ = 0.14).

*B* benign, *M* malignant.

*The differences among four groups were analyzed using the one-way ANOVA with Tukey’s post-hoc test, and its multiple comparisons are as follows: ^a^II-B vs. III-B; ^b^II-B vs. III-M; ^c^II-B vs. V/VI-M; ^d^III-B vs. III-M; ^e^III-B vs. V/VI-M; ^f^III-M vs. V/VI-M.

**Figure 5:**
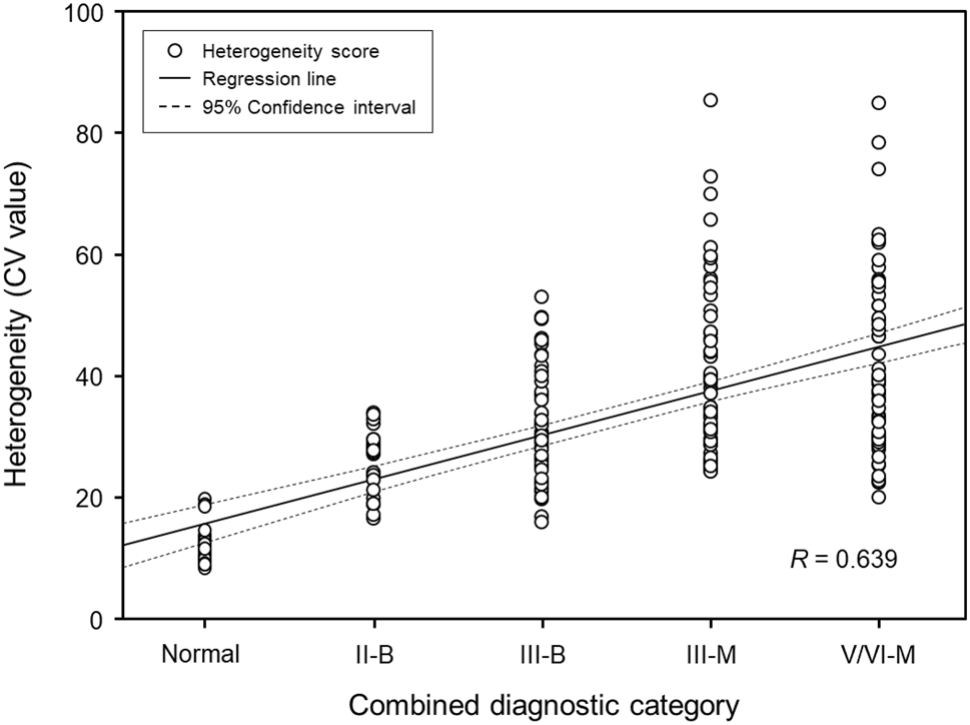
The correlation between heterogeneity scores and combined pathological grades was assessed using a linear polynomial correlation. The graph shows a positive correlation between the heterogeneity scores and degree of malignancy in the pathological grades (*R* = 0.639; *p* < 0.001). The straight line in the plot indicates the linear regression line with 95% confidence intervals (dashed line), and the open circles represent the heterogeneity scores for each patient.

Figure 5 presents the correlations between heterogeneity scores and pathological grades. Heterogeneity scores showed a positive correlation with the degree of malignancy in the pathological grade based on the Bethesda system and surgical pathology (*R* = 0.639, *p* < 0.001).

### ROC analysis for differential diagnosis according to pathological grade

**Figure 6:**
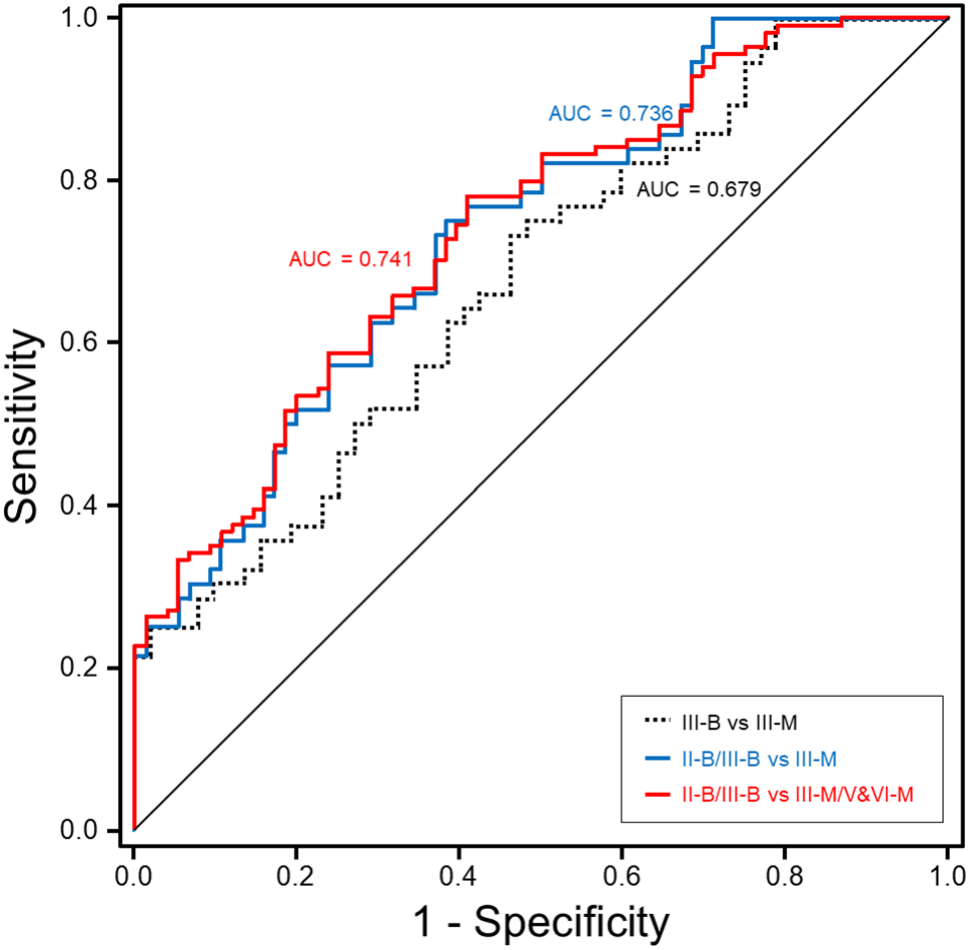
ROC curves of heterogeneity scores for differentiating between benign and malignant nodules. The AUROC curve was 0.679 (95% CI 0.579–0.778) for III-B vs. III-M, 0.736 (0.652–0.820) for II-B/III-B vs. III-M, 0.741 (0.672–0.811) for II-B/III-B vs. III-M/V&VI-M. For the highest diagnostic performance, II-B/III-B vs. III-M/V&VI-M had a cut-off for heterogeneity scores greater than 31.97%, a sensitivity of 0.658, and a specificity of 0.658.

The area under the receiver operating characteristics curve (AUROC) of the heterogeneity scores for the differentiation of pathological grades and their diagnostic accuracy are summarized in Table 3. The mean heterogeneity scores showed good diagnostic performance in differentiating between malignant and benign nodules, with values of 0.736 (0.652–0.820) for II-B/III-B vs. III-M; 0.741 (0.672–0.811) for II-B/III-B vs. III-M/V&VI-M; 0.814 (0.751–0.878) for normal/II-B/III-B vs. III-M; 0.818 (0.764–0.872) for normal/II-B/III-B vs. III-M/V&VI-M (*p* < 0.001, Fig. 6). Among these, the heterogeneity scores showed the highest diagnostic performance in differentiating between the benign (II-B/III-B) and malignant groups (III-M/V&VI-M).

**Table 3:** Receiver operating characteristic curve analysis for diagnosing benign and malignant nodules using heterogeneity scores.

Comparison	Threshold Value	Sensitivity (%)	Specificity (%)	PPV (%)	NPV (%)	DA (%)	AUROC	*p*-value
III-B vs. III-M	33.20	60.7 (34/56)	61.5 (32/52)	63.0 (34/54)	59.3 (32/54)	61.1 (66/108)	0.679	< 0.001
III-B vs. III-M/V&VI-M	33.20	61.4 (70/114)	61.5 (32/52)	77.8 (70/90)	42.1 (32/76)	61.4 (102/166)	0.686	< 0.001
II-B/III-B vs. III-M	31.90	66.1 (37/56)	65.8 (50/76)	58.7 (37/63)	72.5 (50/69)	65.9 (87/132)	0.736	< 0.001
II-B/III-B vs. III-M/V&VI-M	31.97	65.8 (75/114)	65.8 (50/76)	74.3 (75/101)	56.2 (50/89)	65.8 (125/190)	0.741	< 0.001
Normal/II-B/III-B vs. III-M	30.72	73.2 (41/56)	73.1 (79/108)	58.6 (41/70)	84.0 (79/94)	73.2 (120/164)	0.814	< 0.001
Normal/II-B/III-B vs. III-M/V&VI-M	30.22	72.8 (83/114)	72.2 (78/108)	73.4 (83/113)	71.6 (78/109)	72.5 (161/222)	0.818	< 0.001

Data in parentheses are the raw data used to calculate percentages. AUROC, area under the receiver operating characteristic curve.

*DA* diagnostic accuracy = (TP + TN)/(TP + FP + TN + FN), *F* fibrosis stages, *FN* false negative, *FP* false positive, *NPV* negative predictive value, *PPV* positive predictive value, *TN* true negative, *TP* true positive.

The diagnostic accuracy of the mean heterogeneity scores for discriminating thyroid nodule malignancy was 65.9% (87/132) for II-B/III-B vs. III-M; 65.8% (125/190) for II-B/III-B vs. III-M/V&VI-M; 73.2% (120/164) for normal/II-B/III-B vs. III-M; and 72.5% (161/222) for normal/II-B/III-B vs. III-M/V&VI-M.

## Discussion

This study represents heterogeneity scores of the thyroid nodule on the US images and their diagnostic accuracy including correlations with FNAC findings. The quantification of heterogeneity on US images as a part of a morphologic assessment showed good diagnostic performance. Our finding demonstrated that differentiation in the heterogeneity score of thyroid nodule was close association with malignancy possibility. Therefore, it would be provided additional diagnostic information as a promising tool for thyroid nodules with ambiguous FNAC results like AUS/FLUS.

In the clinical practice, the decision to further evaluate a thyroid nodule is frequently encountered. While some thyroid nodules are diagnosed as malignant and require additional assessment, such as in the case of AUS/FLUS thyroid nodules, the majority of these nodules are benign^4^. Thyroid nodules that require surgical treatment are those under suspicion for malignancy or that are malignant or with the induction of hyper-function or compressive symptoms. US is a readily available first-line diagnostic imaging tool for the initial evaluation of thyroid nodules and follow-up of patients undergoing cancer management. TI-RADS suggests candidates for tissue sampling of thyroid nodules based on their composition, echogenicity, shape, margin, and echogenic foci^2^. Based on the US image pattern, location, and size of the nodules, FNAC is commonly performed as a minimally invasive and easily applicable procedure. The interpretation of FNAC findings is categorized according to the potential risk of malignancy using the Bethesda System for Reporting Thyroid Cytopathology^18^. Management of patients with category II or VI thyroid nodules according to the Bethesda system is clear and comprises regular follow-ups and operation. Additionally, patients with category V thyroid nodules undergo total thyroidectomy or lobectomy as standard management, regardless of the range of malignancy risk. However, managing category III and IV nodules poses a challenge for further management options. Diagnostic lobectomy is a conventional management approach for category IV thyroid nodules owing to the difficulty of distinguishing between benign and malignant findings using FNAC. When diagnostic surgery is performed for category III thyroid nodules that are known to be AUS/FLUS, the vast majority (up to 80%) are confirmed as benign^19^. Furthermore, surgical-related complications, such as hypothyroidism, transient or permanent hypoparathyroidism, voice alteration caused by recurrent laryngeal nerve damage, and cosmetic problems, remain as issues despite the surgeon’s experience. As a result, numerous efforts have been undertaken to predict the risk of malignancy in AUS/FLUS thyroid nodules before surgical intervention^20,21^. As mentioned, the diagnosis of thyroid nodules or cancers is a time-consuming process; thus, various computer-aided diagnostic approaches have been introduced to overcome this problem. One study reported that using a machine learning algorithm showed similar results to those of a radiologist’s interpretation when distinguishing between benign and malignant thyroid nodules^13^. Additionally, a deep convolutional neural network model has been shown to improve diagnostic accuracy for thyroid cancer.^14^ Management strategies using various analyses of US images are gradually utilized to improve diagnosis rates and supplement FNAC. For instance, a nomogram using US features and clinical factors has been developed to predict the malignancy of AUS/FLUS thyroid nodules^22^. Recent studies have shown that texture analysis of US images of thyroid nodules can improve the accuracy of nodule diagnosis^11,12^. Moreover, texture analysis using machine or deep learning can provide valuable information about imaging patterns of classified nodules, including signal, size, edge or shape, and internal structure^13–15^.

In the present study, we employed the technique of heterogeneity analysis as a texture analysis method to extract and quantify the parenchymal signal patterns on US images. The quantification software utilized in this study easily demonstrated pixel-based visualization by calculating a heterogeneity score map, and it took approximately 1 min per a single US image to obtained the heterogeneity map. Interestingly, the results of this study showed that heterogeneity scores were significantly different between benign and malignant nodules and that the measurements of the mean heterogeneity were positively correlated with the combined diagnostic category. In particular, the malignant group (III-M/V&VI-M) had substantially higher heterogeneity scores than the normal and benign groups (normal/II-B/III-B). Moreover, a heterogeneity score of 32% or higher was found to be able to differentiate between malignant (III-M/V&VI-M) and benign nodules (II-B/III-B) according to different echogenicity. These findings suggest that the higher the heterogeneity score of the thyroid nodule, the higher the probability of malignancy. Thus, the heterogeneity quantification on US images can aide in making treatment decisions, such as determining the need for biopsy or surgery. Although the exact mechanism of these results remain unclear, it is believed that normal thyroid tissue and benign and malignant thyroid nodules differ in their histological differentiation and cell proliferation, which can affect the morphology and arrangement of tissue cells. The pixel-based heterogeneity maps are capable of reflecting the intrinsic characteristics of thyroid nodules, including normal thyroid tissue, abnormal or uncontrolled growth of cells, neoplasia, and especially heterogeneously malignant areas (Fig. 3). Therefore, it is assumed that normal thyroid tissue and benign and malignant thyroid nodules show variation in heterogeneity scores. In addition to ensuring optimum diagnostic performance of the heterogeneity score in distinguishing between benign and malignant thyroid nodules, the proposed method has the advantage of being able to quickly and easily measure heterogeneity in clinical practice as a non-invasive diagnostic tool. Therefore, heterogeneity quantification of thyroid nodules can serve as a useful diagnostic tool that provides additional diagnostic information for thyroid nodules with ambiguous FNAC results, such as AUS/FLUS. Further studies are required to validate the reliability in large cohort populations.

This study has some limitations. First, this study employed a retrospective cross-sectional design and was conducted at a single institution. Second, the number of II-B cases was smaller than that of other groups because patients with benign results from FNAC underwent thyroidectomy for cosmetic or symptomatic reasons; however, we compared patients with similar TSH levels and age-range in each group to minimize the potential risk of malignancy^23,24^. Third, there were restrictions to the heterogeneity score for assessing thyroid nodules using US images. The accuracy of the heterogeneity analysis can be affected by the quality of the US images. Interpretation of heterogeneity scores requires expertise in image processing and analysis. Therefore, a standardized protocol for measuring heterogeneity is required to ensure consistency and reproducibility. Fourth, most patients with malignancies according to the Bethesda system had PTC or PTC follicular variants. Thus, this finding may potentially lead to false-positive or true-negative findings due to the effect of the disease population. Fifth, the effects of cross-US modality reproducibility on heterogeneity scores were not considered. Thus, a large-scale multicenter study is required to validate our results.

In conclusion, heterogeneity scores differentiated malignant from benign nodules on thyroid US images. Quantitative heterogeneity measurement derived from an US image would be helpful in rapidly discriminating malignant nodules as a non-invasive diagnostic tool to predict the malignant possibility of thyroid nodules, including AUS or FLUS.

## Methods

### Ethics statement

The retrospective cross-sectional research protocol used in this study was approved by the Institutional Review Board (IRB) of the participating Chonnam National University Hwasun Hospital (No. 2023-010). All methods were performed in accordance with clinical practice guidelines. Written informed consent was waived by the IRB committee owing to the retrospective nature of the study and the analysis of anonymized US data, electronic medical records (EMR), and laboratory and pathological reports.

### Patient population

A retrospective analysis was conducted on patients aged ≥ 20 years old with normal thyroid function test who underwent US-guided FNAC and subsequent surgery at our institution between January 2013 and August 2021. Patients with two or more superposed nodules (n = 93), nodule with large cystic areas, or anterior calcification (n = 16) were excluded from the study, as these factors could interfere with the assessment of posterior nodule contours, acoustic radiation force impulse, and shear wave propagation on US images.^25^ Also, Subjects without surgical pathology (n = 54) and datasets including missing value (n = 5) were excluded.

The results of FNAC were analyzed using the Bethesda scoring system, which classifies nodules into six categories based on their cytological characteristics ranging from (a) Class I: nondiagnostic or unsatisfactory, (b) Class II: benign, (c) Class III: AUS or FLUS, (d) Class IV: follicular neoplasm or suspicious for follicular neoplasm, (e) Class V: suspicious for malignancy, and (f) Class VI: malignant^18^. The histologic confirmation was made by pathologists with expertise in thyroid pathology. A total of 188 patients who underwent both FNAC and surgery were enrolled. The final cohort using the combined diagnostic category of the Bethesda system and surgical pathology was divided into four groups according to pathologic grades as follows: II-B (n = 24), III-B (n = 52), III-M (n = 54), and V/VI-M (n = 58) (Fig. 1). These groups have similar mean age ranges (47.4 ± 12.7 years) and TSH levels (2.07 ± 1.29 mIU/L) to minimize the interactions associated with aging and TSH levels.

### Acquisition of US images

Each nodule was evaluated using the LOGIQ E9 (GE Healthcare, Milwaukee, WI, USA) with both 9 MHz linear transducer and 1 to 5 MHz curvilinear transducer or the EPIQ 7G (Philips Healthcare, Cleveland, OH, USA) with both 12 to 15 MHz linear transducer and 1 to 5 MHz curvilinear transducer. B-mode and color duplex Doppler imaging was conducted using the 12 to 15 MHz linear transducer, while elastography was performed using the 9 MHz linear transducer. The examinations were performed by two physicians with over 10 years of experiences. The imaging findings that were evaluated included the nodule dimensions, ratio of the anteroposterior diameter to the transverse diameter, nodule echogenicity, peripheral halo, and calcification.

### Processing and quantification of US data for heterogeneity assessment

To calculate the heterogeneity of US images, we developed a heterogeneity quantification software using the MATLAB program (R2018a, MathWorks, Natick, MA, USA). The software is a customized post-processing program that operates on the Windows platform (ver. XP or higher; Microsoft, Redmond, WA, USA). Following previous studies, we measured pixel-based heterogeneity on images in the Digital Imaging and Communications in Medicine (DICOM) format^16,26^. The primary algorithm for assessing heterogeneity involved opening the US DICOM data, detecting the region boundary manually, segmenting the region on the US image, quantifying the heterogeneity of the segmented US image, and color-mapping the heterogeneity scores (Fig. 2). A heterogeneity score was calculated as a coefficient of variation (CV) value using Eq. (1), and a heterogeneity map was induced by dividing the CV value by each pixel value in the drawn area using Eq. (2).

1 $$ {\text{Heterogeneity score }} = \frac{{\text{Standard deviation}}}{{{\text{Mean}}}}\,  \times\,  100 $$

2 $$ {\text{Heterogeneity map }} = \frac{{{\text{CV}}}}{{\text{Pixel value}}}\,  \times \, 100 $$

### Heterogeneity analysis in clinical patients

All US images were reviewed on a standard picture archiving and communication system using appropriate window settings. The US images in this cohort were assessed blindly and individually by an experienced surgeon (Y.J.R) and a radiologist (J.W.K), each with over 15 years of experience, using customized quantification software. The readers were blinded to each other’s readings and to their previous readings. B-mode US images were obtained in the DICOM format and stored on a console containing the customized MATLAB-based program. After opening the DICOM images on the software, they selected two or three US images per patient to estimate the mean heterogeneity score. During the quantitative measurement of heterogeneity in US images of thyroid nodules, the physicians, who were blinded to the patient’s clinical and pathological information, performed the procedure by positioning a separate ROI along the thyroid nodule contour and normal area on the selected US images. The overall heterogeneity scores for each patient were calculated as the arithmetic mean scores of the measurements. The highest heterogeneity score was considered indicative of the highest degree of inhomogeneity of thyroid parenchyma (Supplementary Fig. 1).

### Statistical analysis

Heterogeneity scores among the combined diagnostic categories were compared using Statistical Package for Social Sciences (SPSS program version 20, Chicago, IL, USA). The variation in the heterogeneity scores among subgroups was analyzed using one-way ANOVA with Tukey’s post-hoc test. Effect size for this study is calculated as a partial eta squared value, η^2^ ^27^. The interpretations of effect size are as follows: η^2^ < 0.01 indicating a negligible effect, 0.01 ≤ η^2^ < 0.06 indicating a small effect, 0.06 ≤ η^2^ < 0.14 indicating a medium effect, and η^2^ ≥ 0.14 indicating a large effect. The association between the heterogeneity scores (a continuous variable) and pathological grades (a categorical variable) was assessed using the linear polynomial correlation (*R*)^28^. To evaluate the diagnostic performance of the mean heterogeneity scores in discriminating between benignity and malignancy, receiver operating characteristics (ROC) curve analysis was performed to calculate the area under the ROC curve (AUROC), sensitivity, specificity, and diagnostic accuracy. Two-sided *p*-values < 0.05 indicated statistical significance for all tests.

### Supplementary Information

Supplementary Figure 1.

## Data Availability

All anonymized data sources described in this study are available from the corresponding author on reasonable request.
