# Supplementary material for: Differential diagnosis of thyroid nodules using heterogeneity quantification software on ultrasound images: correlation with the Bethesda system and surgical pathology
Source: Sci Rep. 2024 May 4;14:10288. doi: 10.1038/s41598-024-60881-2 (PMC11069538; doi:10.1038/s41598-024-60881-2)
Supplement: Supplementary file 1 — Supplementary Figure 1. [file 41598_2024_60881_MOESM1_ESM.docx]

## Supplemental material

**
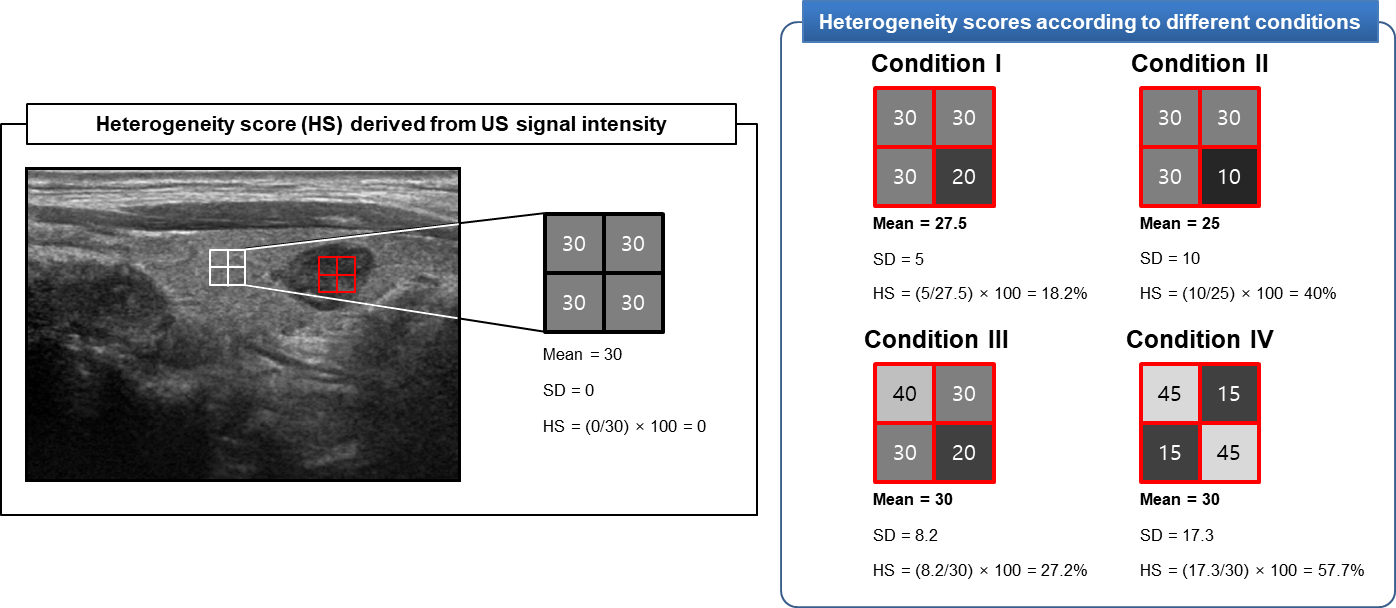
**

**Supplementary Figure 1.** Example of the heterogeneity score (HS) derived from ultrasound (US) signal intensities (left panel). Interpretation of heterogeneity scores according to different conditions (I-IV) within the red region of interest (ROI) (right panel).

HS, heterogeneity score; SD, standard deviation.
